# Simplified screening for the detection of soluble fusion constructs expressed in *E. coli *using a modular set of vectors

**DOI:** 10.1186/1475-2859-4-34

**Published:** 2005-12-13

**Authors:** Annett Dümmler, Ann-Marie Lawrence, Ario de Marco

**Affiliations:** 1 Protein Expression and Purification Facility, EMBL, Meyerhofstr. 1, D-69117, Heidelberg, Germany

## Abstract

**Background:**

The solubility of recombinant proteins expressed in bacteria is often disappointingly low. Several strategies have been developed to improve the yield and one of the most common strategies is the fusion of the target protein with a suitable partner. Despite several reports on the successful use of each of these carriers to increase the solubility of some recombinant proteins, none of them was always successful and a combinatorial approach seems more efficient to identify the optimal combination for a specific protein. Therefore, the efficiency of an expression system critically depends on the speed in the identification of the optimal combination for the suitable fusion candidate in a screening process. This paper describes a set of expression vectors (pETM) designed for rapid subcloning, expression and subsequent purification using immobilized metal affinity chromatography (IMAC).

**Results:**

A single PCR product of two Yellow Fluorescent Proteins (EYFPs) was cloned into 18 vectors comprising identical restriction sites and varying fusion partners as well as differing protease recognition sites. After a small-scale expression, the yields of the different constructs were compared using a Coomassie stained SDS-polyacrylamide gel and the results of this preliminary screening were then confirmed by large-scale purification. The yields were calculated and the stability of the different constructs determined using three independent conditions. The results indicated a significant correlation between the length and composition of non-native amino acid tails and stability. Furthermore, the buffer specificity of TEV and 3C proteases was tested using fusion proteins differing only in their protease recognition sequence, and a His-GST-EYFP construct was employed to compare the efficiency of the two alternative affinity purification methods.

**Conclusion:**

The experiments showed that the set of pETM vectors could be used for the rapid production of a large array of different constructs with specific yield, stability, and cleavage features. Their comparison allowed the identification of the optimal constructs to use for the large-scale expression. We expect that the approach outlined in this paper, i.e. the possibility to obtain in parallel fusion products of the target protein with different partners for a preliminary evaluation, would be highly beneficial for all them who are interested in the rapid identification of the optimal conditions for protein expression.

## Background

Several factors can contribute to poor expression levels or low yields of soluble recombinant proteins produced in *E. coli *[1]: codon bias, lack of post-transcriptional modifications, incorrect membrane targeting, high protease activity, missing partners for complex formation, mRNA instability, limiting redox conditions or chaperone availability. A large number of modified strains is now commercially available which either exhibit a compensative expression of rare codons, co-express specific molecular chaperones, lack protease activity or provide an oxidizing cytoplasm. Furthermore, polycistronic vectors allow the co-expression of several complex subunits and fused leader peptides enable the translocation of ribosomal products to the oxidizing periplasm. However, the most common approach to improve the solubility of recombinant proteins is their fusion with highly soluble partners.

For instance, NusA has been initially selected for being the most soluble protein in *E. coli *[2]. Several other proteins have been proposed as fusion partners [3] but only few of them are commonly exploited. Among them, thioredoxin (Trx) [4], glutathione S-transferase (GST) [5] and maltose binding protein (MBP) [6] offer the further advantage of being suitable for affinity purification, whereas DsbA and DsbC [7,8] are enzymes that facilitate the formation of disulphide bonds. Furthermore, we showed that thermostable proteins improve the yields and enable the purification of the recombinant fusion by simple heat incubation [9].

Nevertheless, some shortcomings are well documented. The linker between GST and the target protein is often instable, leading to the breakage and loss of the target protein [10,11], whereas the solubility of the fusion protein does not yet indicate that the target protein is correctly folded. Soluble aggregates of fusion proteins have been described for both MBP [12] and GST [13,14].

For several applications, the fusion carriers must be removed before using the target proteins. Specific protease recognition sequences are engineered as linkers between the fusion partners and proteolytic digestion used to remove the carriers. Among commercially available proteases, TEV [15] and HRV 3C [16] are the most reliable ones because of their longer recognition site and, consequently, extremely limited non-specific cleavage.

Previous experiments indicate that the optimal conditions to maximize yields, correct folding, and protease cleavage efficiency are protein specific. Therefore, a preliminary screening for the identification of the most suitable expression conditions for the production of soluble proteins is necessary before moving to large-scale production [17]. Approaches that allow the parallel cloning in different vectors simplify the comparison among fusion partners. In this paper we present the results obtained cloning a unique PCR product into the pETM expression vectors developed in our institute.

## Results and Discussion

### General features of the pETM vectors

**Figure 1:**
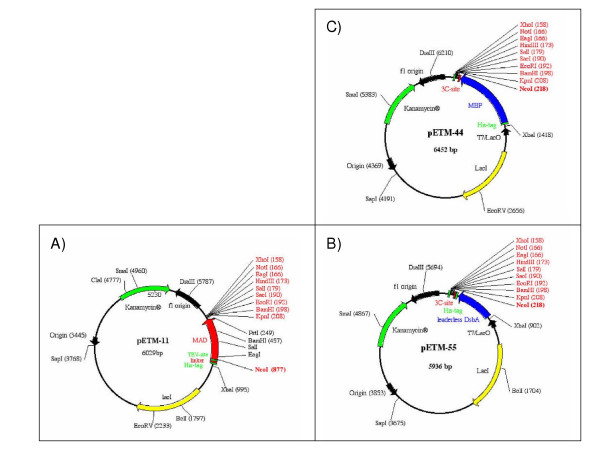
Maps of the pETM vectors. The vectors pETM11, 44, and 55 are shown as representative examples of the different variables used to combine tags and protease recognition sites.

The pETM vectors are derived from pET (Novagene) backbones. They share some common features, the most important of which being a 6xHis tag, a protease recognition site and the conserved multiple cloning site (MCS) starting with an NcoI recognition site (Fig. 1). The NcoI recognition sequence has an in-frame ATG codon that can be used for the functional expression of the target protein, minimizing the number of non-native amino acids at the N-terminus. The conserved MCS ensures that the same couple of restriction sites inserted in the PCR product can be used for direct subcloning in all of the other pETM vectors.

The 6xHis tag suitable for immobilized metal affinity chromatography (IMAC) purification and a protease recognition site, with the exception of the pETM10, are present in all vectors. Both, the TEV and the 3C recognition site versions exist for each vector subclass. The vectors differ in their tag(s) and they are identified by the first number after the pETM code. In particular, 1 indicates the class of pETM vectors with only a His-tag, 2 those with His-tag plus Trx, 3 the combination His-tag and GST (a complete list of the vector maps is available -see additional file 1: "The maps of the pETM vectors"- and their sequences can be downloaded at our website [18]). The second number identifies the specific version that can differ from the others because of the protease restriction site, the relative position of the His-tag at the N or C-terminus of the fusion partner (Fig. 1B and 1C) and the presence of a leader peptide for periplasmic targeting (Table 1). A stuffer gene has been cloned in some vectors (Figure 1A) to simplify the evaluation of the restriction enzyme digestion efficiency.

**Table 1:** Specific features of the vectors. The vectors used in the experiments have conserved cloning sites but differ in the position and identity of the tags and in the protease recognition sequences. Both the EYFP constructs were cloned in all the indicated vectors. (*italic*) functional tag; (*italic*) purification tag;(bold) double-function tag.

**pETM Vectors**	**Composition of the expressed recombinant fusion proteins**			
**10**	*His-Tag*	**EYFP**		
**11**	*His-Tag*	TEV-site	**EYFP**	
**14**	*His-Tag*	C3-site	**EYFP**	
**20**	***Trx***	*His-Tag*	TEV-site	**EYFP**
**22**	***Trx***	*His-Tag*	C3-site	**EYFP**
**30**	*His-Tag*	**GST**	TEV-site	**EYFP**
**33**	*His-Tag*	**GST**	C3-site	**EYFP**
**44**	*His-Tag*	**MBP**	C3-site	**EYFP**
**50**	***DsbA***	*His-Tag*	TEV-site	**EYFP**
**52**	***Ll DsbA***	*His-Tag*	TEV-site	**EYFP**
**54**	***DsbA***	*His-Tag*	C3-site	**EYFP**
**55**	***Ll DsbA***	*His-Tag*	C3-site	**EYFP**
**60**	***NusA***	*His-Tag*	TEV-site	**EYFP**
**66**	***NusA***	*His-Tag*	C3-site	**EYFP**
**70**	**CBP**	TEV-site	**EYFP**	*His-Tag*
**80**	***DsbC***	*His-Tag*	TEV-site	**EYFP**
**82**	***Ll DsbC***	*His-Tag*	TEV-site	**EYFP**

Only pETM20 carries the β-lactamase gene that confers ampicillin-resistance, whereas all the other vectors are kanamycin-selectable.

### Comparison of the constructs

Two EYFPs (wild type and I48A mutant) were cloned into the pETM vectors. The specific features of the constructs used in the analysis are listed in Table 1. The His-tag is directly fused to the target protein in the pETM10 vector whereas a TEV and a 3C recognition site is inserted between the target protein and the tag in vectors pETM11 and 14, respectively. The pETM20 and 22 vectors contain the stabilising Trx fusion tag [4] and differ only in the protease restriction site. Cloning into pETM30 and 33 vectors allows the expression of GST-fusion proteins that can be purified by two independent and alternative affinity methods. Also, the MBP-EYFP fusion protein obtained by cloning into the vector pETM44 can be purified by both, maltose affinity and IMAC. In the case that the correct folding of recombinant proteins involves the formation of disulfide bonds the yield can be improved by fusion with DsbA and DsbC, both in the cytoplasm [8] or after targeting into the oxidizing environment of the periplasm. The vectors pETM50, 52, 54, 55, 80, and 82 cover all of these possibilities. The pETM60 and 66 vectors allow the fusion of the target protein with the highly soluble bacterial NusA protein [2], and the pETM70 leads to the expression of fusion proteins with the calmodulin-binding protein.

The yield of soluble EYFP expressed using the commercial pRSET vector (Invitrogen) was used as a reference. The crucial features for expression control (origin of replication, promoter) of pRSET and pETM are the same, but they differ in the length of the region between the His tag and target protein.

### Small-scale expression screening

Small-scale (1.5 mL) cultures contain enough material for a rapid comparison of recombinant protein yields. The bacterial lysates from wild type and mutant EYFP were analysed by SDS-PAGE and used to estimate the total expression levels of the recombinant constructs while the soluble fractions recovered after centrifugation were used to purify the recombinant proteins by IMAC using Talon resin. We found this information more reliable than the analysis of the total soluble fraction in which soluble aggregates can be highly represented [12-15,19].

**Figure 2:**
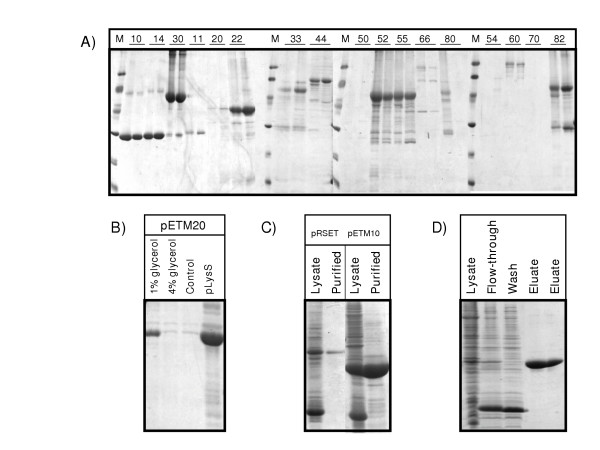
Comparison of the wt and I48A mutant EYFP soluble yields using the different pETM vectors. A) Small-scale affinity purification of two EYFPs (wild type and mutant I48A) expressed in BL21(DE3) bacterial cells and using the following vectors: pETM10, pETM14, pETM30, pETM11, pETM20, pETM22, pETM33, pETM44, pETM50, pETM52, pETM55, pETM66, pETM80, pETM54, pETM60, pETM70, pETM82. B) Small scale affinity purification of constructs expressed by pETM20 under different growth conditions and the alternative bacterial strain BL21(DE3) pLysS. C) Small-scale purification of wt EYFP expressed in pRSET and pETM10 vectors. D) Large-scale purification pattern of wt EYFP expressed in pETM11.

All of the vectors expressed recombinant proteins of the expected molecular mass but the amount of accumulated recombinant proteins differed among the vectors: GST fusion>His only>MBP fusion>DsbA fusion>DsbC fusion = NusA fusion>Trx fusion. Apparently, the expression rate is dependent on the fusion partner because similar yields were obtained expressing the fusion partners alone: GST = MBP>DsbC>DsbA = NusA>Trx (data not shown), although the regulative expression elements are the same for all the vectors. Codon bias, mRNA or protein stability [20] can further influence the level of protein accumulation. For instance, wild type and mutant EYFP expressed using the same vector often differed for the amount of accumulated soluble protein (Fig. 2A). The comparative screening indicated that the mutant EYFP was more soluble than the wild type when expressed from the pETM22 and 33, whilst the wild type when expressed from the pETM66.

SDS-PAGE analysis of the IMAC purified recombinant proteins from bacteria transformed with the different vectors is shown in Figure 2A. EYFP depends on a reducing environment to successfully complete its folding and its expression in the periplasm was used as a negative control. As expected, the recombinant proteins secreted in the periplasm expressed from pETM50, 54, and 80 were mostly not soluble. A negative effect of the fusion partners DsbA and DsbC is ruled out since the leaderless versions of the same vectors (pETM52, 55, and 82) largely accumulated in the soluble fraction.

All of the remaining constructs were soluble, with the exception of the calmodulin-binding fusion (pETM70). The amount of purified proteins seemed strongly vector-dependent but it is necessary to remind that this comparison is rather qualitative because larger constructs (fusions with MBP or NusA, namely pETM44, 60, and 66) stain less than constructs with smaller fusions (Trx, GST, DsbA, and DsbC: pETM20, 22, 30, 33, 52, 55, 82) or no fusion partners (pETM10, 11, 14).

A second round of screening can be envisaged in which different expression conditions can be tested in parallel for their effect on the soluble protein yields. For instance, the pETM20 vector is the only ampicillin-resistant and has a different backbone. The constructs from the pETM20 were poorly expressed and gave a low yield of soluble protein when standard growth conditions were used. The metabolization of the ampicillin could result in the loss of selectivity for the transformed bacteria and their substitution in the liquid culture with wild type cells. Furthermore, a higher level of expression leakage can lead to mutations of the T7 polymerase, with consequent decreased transcription functionality and repressed expression of the recombinant protein [21]. Therefore, the pETM20-EYFP vector was cultured using the degradation-resistant carbenicillin instead of ampicillin and either transformed in pLysS cells or cultured in the presence of glycerol to prevent expression leakage. As a result, the yields of the soluble proteins were dramatically increased (Fig. 2B).

Apparently, even minor differences in the sequence of an expressed construct can significantly affect the protein solubility, as in the case of EYFP from pETM11 and pETM14 that differ only in the protease recognition site. We compared two other similar vectors to gain further information about the critical features involved in protein solubility. The EYFP construct from pETM10 accumulated at higher concentrations than that from pRSET (Fig. 2C, total lysates). Furthermore, the first construct was apparently soluble because concentrated by the affinity purification step while the EYFP expressed from pRSET seemed mostly insoluble (Fig. 2C). The presence of a long, not structured region, at the N-terminus of the recombinant protein expressed by pRSET could explain the different results because, otherwise, the two vectors share the same expression regulative features.

The first set of data show that the screening step based on affinity purification and SDS-PAGE analysis is a reliable tool for the identification of soluble recombinant protein, select among constructs expressed from vectors belonging to the same subclass (for instance, pETM10 and 14 with respect to 11), the comparison of different growth conditions, and the evaluation of the degradation rate of the constructs.

### Large-scale purifications

The constructs with the wild type EYFP version were purified from the pellet corresponding to 1 L culture. The supernatants obtained after lysate ultracentifugation were loaded onto CoCl_2_-activated sepharose columns and the recombinant His-tagged protein was eluted after a washing step. After desalting the purified protein was used for stability tests and its concentration calculated according to its specific absorbance at 280 nm (Table 2). A typical purification pattern (EYFP from pETM10) is reported in Figure 2D to show that the non-specific binding is negligible after extensive washing.

**Table 2:** Yields calculated after large-scale purification and stability of the wild type EYFP fusion proteins generated using the different pETM expression vectors. Three different aggregation tests were performed (see M & M) and the symbols indicate: aggregation (+), no aggregation (-), not performed (/).

**Constructs**	**Soluble fusion protein yield (mg/Lculture)**	**Aggregation tests**
EYFP-10	11.5	---
EYFP-11	1.1	-++
EYFP-14	13.8	---
EYFP-20	2.7	---
EYFP-22	4.9	---
EYFP-30Gluthatione	16.6	---
EYFP-30His	23.3	---
EYFP-33His	18.7	---
EYFP-44	4.2	---
EYFP-50	0	/
EYFP-52	26.8	---
EYFP-54	0	/
EYFP-55	23.1	---
EYFP-60	42.2	---
EYFP-66	40.8	---
EYFP-70	0	/
EYFP-80	0	/
EYFP-82	1.4	+++

The highest yields of EYFP fusions were obtained using NusA as a stabilizing partner (constructs 60 and 66) but high yields were also obtained with fusions with both GST and DsbA (30, 33, 52 and 55) and using the vectors pETM10 and 14 (only His tag) (Table 2). The yields of pure EYFP obtained from the pETM10, 14, 60, and 66 become comparable after removal of the carrier proteins. Trx, DsbA, and DsbC, (pETM20, 22, 52, 55, and 82) fusions yielded lower amounts of soluble protein, as also with the pETM11 vector (His tag only). The constructs expressed from pETM11 and 82 became instable after incubation at 30°C overnight or after repetitive freezing and thawing cycles (Table 2). This was in contrast to all of the other proteins with fusions that remained monodisperse after these trials. The large-scale experiments confirmed that no soluble protein could be recovered from bacteria transformed with pETM70 (fusion to calmodulin-binding protein) and vectors expressing constructs for periplasmic secretion (pETM50, 54, and 80).

In some cases the fusion constructs appeared to be partially cleaved at the level of the linker during the purification. Roughly 10% of the protein purified from bacteria expressing DsbA-EYFP fusions was represented by DsbA alone. Similarly, we recovered 15% of pure GST from the GST-EYFP fractions and 35% of DsbC as a degradation product of the DsbC-EYFP fusion (data not shown). These results confirm that observed at small-scale level (Fig. 2A) and indicate that the fragility at the linker position is not a specificity of the GST fusions [11]. Similar degradation products also occur in large scale screening experiments performed using GFP, GST, Trx, MBP as fusion partners [17]. What we observed with other proteins is that the degree of degradation using a specific vector varies from negligible levels to almost 100% [11], suggesting that is the specific interaction carrier-target protein to determine the fragility of the construct rather than the carrier itself.

Our results give an indication of how much the length and composition of the expressed non-native N-terminus domain (His-tag/linker/protease recognition sequence) influence the stability of the expressed constructs. The solubility and yields of EYFP recovered from bacteria transformed with pRSET and pETM11 were low (0.4 mg/mL and Fig. 2; 1.1 mg/L, Table 2) in comparison to EYFP from pETM10 and 14 (more than 10 mg/L, Table 2). The EYFP construct expressed from pRSET has a 33 aa tail at the N-terminus, that of pETM11 has 26 extra aa while the EYFP from pETM14 has only 18 aa and the one from pETM10 has as few as 9. Furthermore, the pETM14 linker has more flexible glycine residues and less large aa than the linker in pETM11. EYFP obtained after proteolytic digestion of any of the fusion constructs was soluble and stable, indicating that the destabilizing effect was specifically due to the extra non-native amino acids at the N-terminal.

A possible explanation for the poor results obtained using the cytoplasmic DsbC-EYFP construct from pETM82 would take into consideration the enzymatic activity of the fusion partner. In fact, DsbC has been shown to be capable of catalyzing the formation of disulfide bonds even in the reducing cytoplasm [8] whilst their formation seems incompatible with a stable structure of EYFP.

We were also interested in comparing some other parameters. The position of the His-tag at the N-terminus (GST and MBP fusions, Table 1) did not apparently improve the recovery of the recombinant fusions during the affinity purification. In fact, the yields of constructs in which the His-tag is expressed in the theoretical less accessible between the fusion partner and the EYFP -as for NusA and DsbA fusions- were comparable (Table 2) and no more unbound recombinant protein was detected in the flow-through and wash fractions (data not shown).

We also used the double tagged GST-EYFP to compare the purification efficiency of ion metal affinity for 6xHis and glutathione sepharose for GST. The yield of the fusion purified using the His-tag was slightly higher (Table 2) but the higher affinity for GST using glutathione-sepharose resulted in less non-specific impurities. The amount of contaminants due to degradation was in the same proportions (data not shown).

### Specificity of the protease cleavage site

**Figure 3:**
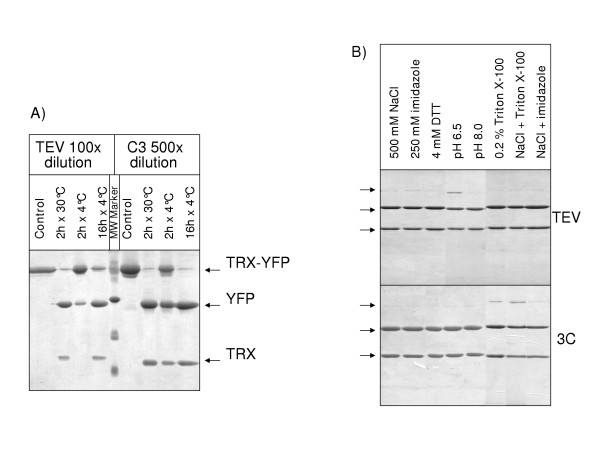
Comparison of the protease efficiency of TEV and C3. A) The Trx-EYFP fusion protein recovered using the pETM20 and pETM22 vectors was digested in the presence of 1 μg (TEV) and 0.2 μg (C3) protease for 100 μg of fusion protein. B) The Trx-EYFP constructs resuspended in different buffers were digested at 30°C for 3 h in the presence of 1:100 (TEV) and 1:500 (3C) diluted proteases.

The protease recognition sequence had no significant influence on the protein solubility. In fact, the constructs expressed by the TEV and 3C versions of the same vectors (pETM20 and 22, 30 and 33, 60 and 66) gave similar yields (Table 2). The cleavage efficiency of TEV and 3C was compared using EYFP-Trx fusions from pETM20 and 22 as substrates. Both proteases cleaved more than 95% of their substrates after incubation at 30°C for 2 h (Fig. 3A). Nevertheless, 3C could be used 5 times more diluted than TEV, namely 2 μg of protease for mg of substrate instead of 10 μg. Furthermore, 3C was confirmed to be more active than TEV at 4°C (Figure 3A and [16]). The smaller mass of 3C in comparison to TEV could lead to a decreased steric hindrance and allow the facilitated access to the recognition site [22]. Otherwise, both proteases remained active in buffers containing up to 500 mM NaCl, 250 mM imidazole, and 4 mM DTT (Fig. 3B). 3C was more sensitive to detergents (Triton X-100 and Brij 58) than TEV but less pH-dependent. Most of the combinations of salt, detergent and imidazole had no synergic inhibitory effect (Fig. 3B). In conclusion, the choice of either of the two proteases allows the selection of a cleavage buffer which is at least partially optimized for the stability of the substrate protein.

## Conclusion

Comparison of the yields of the purified EYFP constructs from the different expression vectors indicated that fusions with NusA and GST, as well as constructs with His-tags and short linkers, resulted in the highest yields of soluble recombinant protein. Furthermore, we did not observe better affinity binding using constructs with a His-tag at the N-terminus than with constructs with an internal tag and the His-GST-EYFP was purified at similar quantitative and qualitative levels using any of the two affinity tags. The analysis of the aggregation state of the purified proteins showed that most of the constructs were both soluble and monodisperse [23].

The solubilizing effect of the different tags has often been reported but independent experiments are difficult to compare because they were performed using a great variety of growth and detection conditions and often with a limited number of test proteins [6,24,25]. More systematic investigations [17] and our experience involving the expression of hundreds of proteins using pETM vectors suggest that the best tag and growth conditions are protein specific. Therefore, the aim is not the identification an improbable ideal tag that always ensures better solubility to any fused passenger protein but to develop strategies for fast and easy screening among different vectors before starting the large-scale production. The pETM collection facilitates the sub-cloning and simplifies the result comparison. With respect to other modular systems used in high throughput proteomics and based on ligation independent cloning [26] and recombination [27,28] rather than classical cloning, the pETM vectors can provide shorter extra sequences at both protein termini. The negative effect of extra amino acids and poorly structured regions on the protein solubility has been related to their length and composition [29-33] and confirmed by our experiments performed with the constructs expressed by pRSET, pETM10, 11, and 14. Finally, the pETM vectors offer the choice of the specific protease for removal of the tags thus widening the opportunity to select buffer conditions more compatible with the stability of the target protein (Fig. 3).

## Methods

### Cloning into the pETM vectors

Two EYFP (Yellow Fluorescent Protein, AAU85108) sequences (wild type and mutant I48A) cloned into pRSET (Invitrogen) were used as a template for the PCR reactions. The PCR primers were designed to contain the NcoI restriction site (forward) and either the EcoRI or the NotI (reverse). Two series of pETM vectors [18] were digested with NcoI/EcoRI and NcoI/NotI, respectively, and the digested PCR products were ligated both overnight and by quick-ligation. The four parallel ligation products for each vector were used to transform DH5α competent cells and the highest efficiency was obtained using the combination NcoI/EcoRI and overnight ligation. After plasmid amplification and sequencing, the pETM expression vectors containing the sequence-verified EYFP insert were transformed in BL21 (DE3) competent cells and the colonies used to inoculate 3 mL of LB for preparing bacterial glycerol stocks and material for the small-scale screening.

### Bacterial culture and protein purification

The bacteria were grown at 37°C until they reached an OD_600 _of 0.4, the temperature was then decreased to 20°C, the recombinant expression induced with 0.1 mM IPTG at an OD_600 _of 0.6 and the pellet corresponding to 1.5 mL was recovered after overnight culture and stored at -20°C. Frozen pellets were resuspended in 350 μ L of 20 mM TrisHCl buffer, pH 8.0, (metal affinity purification) or 350 μ L of 20 mM PBS buffer, pH 7.3, (glutathione sepharose purification) containing 5 mM MgCl_2 _1 mM PMFS, 1 mg/mL lysozyme, and 1 μ g/mL DNase. NaCl was added to the Tris buffer to a final concentration of 300 mM. The samples were sonicated for 5 min in a water bath and the total lysate samples were taken before centrifugation at 5,000 g for 10 min. The resulting supernatants were added to either 25 μ L of Talon beads (Clontech) equilibrated in 20 mM Tris buffer, 300 mM NaCl, or 25 μ L glutathione Sepharose 4 Fast Flow resin suspension (Amersham) equilibrated in 20 mM K-phosphate buffer, 150 mM NaCl, respectively. After incubation with shaking at room temperature for 30 min the affinity matrixes were recovered by a short centrifugation and the supernatant discarded. The glutathione sepharose resin was resuspended in PBS, washed for 30 min, recovered as above and then washed again before being resuspended in 25 μ L of SDS-PAGE loading buffer. The Talon beads were washed twice for 30 min in 20 mM Tris-HCl, pH 8.0, 500 mM NaCl, 20 mM imidazole and finally recovered in 25 μ L of SDS-PAGE loading buffer. The purified proteins were visualized by SDS PAGE.

The purification protocols were modified at the following steps when large-scale purifications were performed. Pellets from 1L bacterial culture were re-suspended in 4 volumes of buffer (PBS + 5 mM MgCl_2 _and 1 mM PMFS or 20 mM TrisHCl buffer, pH 8.0, 500 mM NaCl, 10 mM imidazole 5 mM MgCl_2 _and 1 mM PMFS) and sonicated 5 min in a water bath. 1 mg/mL lysozyme and 1 μ g/mL DNase were added and the lysates were incubated with shaking at room temperature for 30 min. The samples were centrifuged at 90.000 × g for 35 min, the supernatants filtered and loaded onto a pre-equilibrated GSTrap or Hi-Trap chelating columns (Amersham) charged with CoCl_2_, respectively, connected to a FPLC system. The elution was performed using either 25 mM Tris HCl, pH 8.0, 10 mM reduced glutathione (glutathione affinity) or 20 mM Tris HCl, pH 8.0, 250 Mm imidazole (IMAC). The purified proteins were buffer exchanged into 50 mM PBS containing 10% glycerine using a HiTrap Desalting column (Amersham), the protein concentration was calculated after measurement of the absorbance at 280 nm, and samples were prepared for SDS-PAGE.

### Protein aggregation analysis and protease activities

Protein aggregation was measured according to Nominé et al. [34] using a luminescence spectrometer (SLM Aminco). The stability of the purified constructs was determined by comparing the aggregation index calculated using fresh samples (control) with: a) samples thawed after incubation at -80°C for 24 hours, b) samples frozen and thawed for 5 cycles, c) incubated at 30°C for 24 hours.

Protein gels were stained using SimplyBlue Safe Stain (Invitrogen) and the relative protein content in each band quantified using the public domain NIH Image program (developed at the U.S. National Institutes of Health [35]).

TEV and 3C proteases were used at concentrations varying from 1:100 to 1:1000 dilutions with respect to the substrate, at 4 and 30°C, and using 2 or 18 hour incubation. The different buffers are indicated in the legend to the Figure 3.

**Additional File 1**. (PDF) "The maps of the pETM vectors". The maps and the MCS regions of the pETM vectors used in the experiments are reported.
